# Study protocol: Exploratory trial of Forza™, an osmotin-based nutraceutical as adjuvant for the treatment of progressive multiple sclerosis

**DOI:** 10.1371/journal.pone.0311214

**Published:** 2025-02-27

**Authors:** Viola Costa, Kenda Aluan, Irene Schiavetti, Caterina Bason, Tiziana Vigo, Elisa Leveraro, Corrado Cabona, Valeria Prada, Mauro Costagli, Vincenzo Daniele Boccia, Bruno Ruggiero, Giampaolo Brichetto, Marco Salvetti, Maria Pia Sormani, Gianluigi Mancardi, Matilde Inglese, Mario Alberto Battaglia

**Affiliations:** 1 Department of Neurosciences, Rehabilitation, Ophthalmology, Genetics, Maternal and Child Health (DiNOGMI), University of Genoa, Genoa, Italy; 2 Department of Health Sciences, Section of Biostatistics, University of Genoa, Genoa, Italy; 3 IRCCS, Ospedale Policlinico San Martino, Genova, Italy; 4 IRCCS, Ospedale Policlinico San Martino, UOC Neurofisiopatologia, Genoa, Italy; 5 Italian Multiple Sclerosis Society Foundation (FISM), Scientific Research Area, Genoa, Italy; 6 9th Dimension Biotech, Inc, McMinnville, Oregon, United States of America; 7 Associazione Italiana Sclerosi Multipla (AISM) Rehabilitation Center, Genoa, Italy; 8 Centre for Experimental Neurological Therapies (CENTERS), Department of Neurosciences, Mental Health and Sensory Organs, Sapienza University, Rome, Italy; 9 IRCCS Istituto Neurologico Mediterraneo Neuromed, Pozzilli, Italy; Karolinska Institutet, SWEDEN

## Abstract

**Background:**

Multiple Sclerosis (MS) is the first cause of non-traumatic neurological disability in young adults. Primary and secondary progressive MS are still lacking effective treatments. A new nutraceutical product made of lyophilised leaves of bioengineered kiwi plants (*Actinidia deliciosa*) overexpressing osmotin has recently been developed. Osmotin is a protein associated with stress adaptation in plant cells and it shares anti-inflammatory and neuroprotective properties with mammalian adiponectin. The aim of this study is to explore the safety and the efficacy of osmotin in progressive MS (PMS).

**Methods:**

This is a prospective, multicenter, single-arm interventional, baseline vs treatment study that will be carried out by two Italian MS centers, where a total of fifty PMS patients will be recruited. Every patient will take a daily dosage of 5 grams of an osmotin-based nutraceutical, named Forza™ (9^th^ Dimension Biotech, Inc.), for 6 months. Two pre-treatment assessments, at -6 months (-6M) and at baseline visit (M0), and two post-treatment assessments, at month 1 (M1), and at month 6 (M6) will be carried out. Forza^TM^ safety and activity, assessed by serum Neurofilaments Light (NfL) Chain quantification, are the primary outcomes of the study. Additional assessments will consist of clinical and neuropsychological evaluations, patient reported outcomes (PROs), brain magnetic resonance imaging (MRI), motor evoked potentials (MEPs) and optical coherence tomography (OCT).

**Discussion:**

Disease modifying treatments in MS usually target inflammatory pathways with excellent results on reducing relapse associated disability but fail in preventing progression independent from relapse activity. This is a proof-of-concept study aimed at exploring the safety and the activity of an osmotin-based nutraceutical as an adjuvant treatment in PMS patients.

**Trial registration:**

The trial was registered on July 10^th^ 2023 at www.clinicaltrials.gov having identifier NCT05937802.

## Introduction

Multiple sclerosis (MS) is the first cause of non-traumatic neurological disability in young adults [1]. In relapsing remitting MS (RRMS) disease modifying treatments (DMTs) successfully prevent relapse associated worsening (RAW) [2] by reducing the occurrence of relapses. In progressive MS (PMS) disability accumulates mainly without occurrence of clinical relapses and is characterized by progression independent from relapse activity (PIRA) [2]. Different pathophysiologic mechanisms seem to interact, resulting in myelin damage and neurodegeneration through incompletely understood events [3]. Therefore, while targeting inflammatory pathways has advanced the efficacy of treatments in RRMS, progressive forms lag behind [4]. Moreover, disability progression in MS occurs irrespective of the disease phase: PIRA can manifest during both the RR and the progressive phase [5, 6]. Consequently, a notable proportion of individuals with RRMS, despite receiving the most effective therapies, may still transition to secondary progressive MS (SPMS) [7]. To prevent or limit disease progression, various nutraceutical interventions that target specific pathways implicated in MS pathology (such as inflammation, oxidative stress, and mitochondrial dysfunction) have been trialed for their efficacy as add-on treatment in MS [8].

Osmotin is a protein associated with stress adaptation in plant cells [9]. Actinidia Deliciosa plants overexpressing the osmotin protein were developed at the University of Tuscia in Italy and tested for ten years for their improved agronomic and nutritional qualities [10]. The anti-inflammatory effects of Actinidia plants are well-documented [11] and osmotin gene overexpression has been shown to increase the production of flavon-3ols and other anti-inflammatory plant compounds [12]. In mammals, osmotin binds the adiponectin receptor due to the 3D structure homology between the two proteins [13]. As such, it may share some of the anti-inflammatory effects of adiponectin [14–16], such as the attenuation of LPS-induced neuroinflammation with preventive effects on vascular inflammation in atherosclerosis [15–17]. Osmotin also protects against glutamate-induced synaptic dysfunction and neurodegeneration in the rat brain [16]. However, adiponectin and its isoforms also have pro-inflammatory functions [18], that may not be shared by osmotin because of the different amino acid sequence other than the active site [19].

Several studies have investigated the effects of osmotin on synaptic dysfunction and neurodegeneration in vivo and in vitro. For instance, a study examined the effect of osmotin on cell cultures and mice subjected to glutamate-mediated excitotoxicity: in vitro, osmotin treatment was found to reduce glutamate-mediated DNA free radical production and decreased cell viability and cytotoxicity induced by glutamate. In vivo, mice hippocampus was analyzed, and osmotin treatment was found to reduce DNA damage and cell death mediated by glutamate excitotoxicity; moreover, it restored the localization and distribution of proteins necessary for synaptic function [20].

In another investigation, the effect of osmotin on in vitro cell coltures and in vivo models of Parkinson’s disease (PD) was investigated, exploiting the phosphorylation of 5′ adenosine monophosphate-activated protein kinase (AMPK) via adiponectin receptor 1 (AdipoR1), thus attenuating PD-associated pathology. According to this study, osmotin mitigated MPTP- and α-synuclein-induced motor disfunction and it reduced neuronal cell death and neuroinflammation by regulating the mitogen-activated protein kinase (MAPK) signaling pathway. Additionally, osmotin alleviated the accumulation of α-synuclein by promoting the AMPK/mammalian target of rapamycin (mTOR) autophagy signaling pathway [21].

In both studies [20, 21], the osmotin dosage was 10 mg/die/kg of the subject’s body weight for a limited period of time, up to a maximum of five weeks.

Based on these premises, a nutraceutical product (commercial name Forza™) has been developed by 9th Dimension Biotech, Inc., consisting of the lyophilised leaves of the Actinidia Deliciosa plants bioencapsulating recombinant osmotin, with the goal of offering the combined benefits of the osmotin protein in conjunction with the enhanced anti-inflammatory properties of the bioengineered Actinidia deliciosa plants to consumers. In our study, Forza™ is administered for a period of six months; therefore, the daily dosage of lyophilized osmotin was adapted from the 10 mg/die/kg used in vitro and in vivo [20, 21] to 0.4 mg/die for an average subject weight of 70 kg.

The bioencapsulation of protein drugs in lyophilized plant cells represents an ideal method of oral protein drug delivery to patients, which surpasses all other methods of protein drug delivery both in safety and efficacy [22]. Since current therapies for PMS are not effective enough to overshadow possible effects of add-on therapies, we will explore the safety and efficacy of Forza™ as an add on therapy in this population.

## Materials and methods

### Aims of the study

The co-primary aims of this study are to verify the safety of Forza™ in PMS during a 6 months treatment period and evaluate its efficacy on disease activity, assessed by the quantification of serum Neurofilaments Light (NfL). The secondary aim is to explore its clinical and biological efficacy in PMS.

### Study design and population

This is a prospective, multicenter, single-arm interventional, baseline vs treatment study that is planned to be conducted at the MS Centers of the San Martino Hospital in Genoa, and of the Sant’Andrea University Hospital in Rome.

A total of fifty (50) PMS patients will be recruited, comprising individuals either untreated or under their current disease-modifying therapy (DMT), with twenty-five (25) from each MS center. The enrolled PMS patients will be preferably in a condition of neurological stability, without any therapy changes in the previous year and throughout the trial. The inclusion criteria consist of: a diagnosis of progressive MS (PMS), age between 18 and 70 years old, an Expanded Disability Status Scale (EDSS) score of ≤ 6.5, and no contraindications to MRI.

Exclusion criteria consist of: any severe concomitant renal, hepatic, oncological, hematological and psychiatric diseases, HIV positivity and pregnancy.

The recruitment period has started on the January 2^nd^, 2023, and it is still ongoing.

### Intervention

The treatment consists in the oral administration of a dosage of 5 grams per day of Forza™, provided in the form of capsules or powder (9^th^ Dimension Biotech, Inc.). Each capsule contains approximately 0.7 grams of Forza™. The dosage of 5 grams per day supplies 27 mg of lyophilized osmotin. The capsules will be administered as follows: 4 capsules in the morning and 3 capsules in the evening for 6 months (S1 Fig).

### Settings and procedures

The study includes two pre-treatment assessments: a first neurological evaluation six months before Forza™ assumption (-6M)—where the appointed doctor will evaluate trial eligibility and obtain signed informed consent—and a subsequent baseline neurological evaluation (M0), right before Forza™ is administered and taken orally by the patients. Two subsequent neurological follow-up evaluations are planned at 1 month (M1) and at six months (M6) of treatment respectively.

At each timepoint neurophysiological tests, Optical Coherence Tomography (OCT) and NfL quantification will be performed. In addition, clinician assessed outcomes, performance measures and patient reported outcomes will be acquired to investigate the following neurological domains:

- Overall disability will be evaluated by Expanded Disability Status Scale (EDSS).
- The motor domain will be evaluated by Timed 25 Foot Walk (T25FW), 12-item Multiple Sclerosis Walking Scale (MSWS12), Nine-Hole Peg Test (9HPT).
- The cognitive domain will be evaluated through Montreal Cognitive Assessment (MoCA) and Symbol Digit Modalities Test (SDMT).
- The emotional domain will be assessed by Hospital Anxiety and Depression Scale (HADS).
- The bladder domain will be assessed by the Overactive Bladder questionnaire (OAB-q).

Brain MRI will be performed at each timepoint. MRI protocol will include axial T2 weighted Turbo Spin Echo (TSE), axial FLAIR, high resolution 3D sagittal T1-weighted sequence, axial diffusion tensor imaging (DTI) sequence. In a subgroup of twenty-five (25) patients enrolled in the MS center of the San Martino Hospital in Genoa, multi-shell diffusion-weighted (DWI) sequence and proton magnetic resonance spectroscopy (1H-MRI) will be performed to investigate the potential effect of Forza™ on brain microstructure and brain metabolism such as glutamate, N-acetylaspartate, creatine and choline concentration changes.

Any changes in disease modifying therapy (DMT), any occurrence of adverse events during treatment period and any interruption of treatment will be recorded on the medical chart.

An overview of the schedule of enrolment, interventions, and assessments at every time point is summarized in Table 1.

**Table 1:** Forza^TM^–Schedule of enrollment, intervention and assessments.

	Pre-treatment -6 months	Pre-treatment 0 month	Follow up +1 month
	**(-6M)**	**(0M)**	**(M1)**
*Demography*	**X**		
*MS diagnosis and history*	**X**		
*Clinical evaluation* *(any changes in therapy*, *any relapses)*		**X**	**X**
*Eligibility screen*	**X**		
*Informed consent signing*	**X**		
*Treatment assumption beginning*		**X** *	
*NfL*	**X**	**X**	**X**
*MEPs*	**X**	**X**	
*OCT*	**X**	**X**	**X**
*EDSS*	**X**	**X**	**X**
*T25FW*	**X**	**X**	**X**
*MSWS12*	**X**	**X**	**X**
*9HPT*	**X**	**X**	**X**
*MOCA*	**X**	**X**	**X**
*SDMT*	**X**	**X**	**X**
*HADS*	**X**	**X**	**X**
*OAB*	**X**	**X**	**X**
*Brain MRI*	**X**	**X**	**X**
*MRI with DWI and 1H-MRI* **	**X**	**X**	**X**
*Treatment compliance*			**X**
*AE collection*			**X**

* Only after the execution of all assessments

**Only for a subgroup of patients

### Outcomes and assessments

#### Primary outcomes

The primary outcome of this study will be the safety of Forza™ as an add-on therapy in PMS assessed through the incidence and severity of treatment-related adverse events after 1 month and 6 months of therapy. The co-primary outcome will be Forza™ efficacy in the reduction of serum NfL level after 6 months of Forza™ assumption as compared to the pre-treatment NfL level.

#### Secondary outcomes

The impact of Forza™ on clinical and neuropsychological assessments, patient reported outcomes (PRO’s), motor evoked potentials, retinal atrophy and brain metabolism and microstructure will be evaluated at each timepoint.

### Study assessments

#### Serum Neurofilament light chain (NfL) quantification

Serum NfLs quantification will be performed using the ELLA Simple PlexTM (ProteinSimple) microfluidic platform, which allows fast and ultra-sensitive quantification of NfLs, with a detection limit of 2.7 pg/ml [23]. Recent studies have verified that NfLs quantification on serum with ELLA Simple PlexTM is equivalent to the current reference method Single Molecular Array (Simoa, Quanterix) [24].

Serum samples will be processed and frozen within 4 hours. Serum NfLs quantification of all the collected samples will be carried out at the end of the study, using ELLA microfluidic plates that allow dosing of 70 samples simultaneously.

Neurofilament light (NfL) chain quantification is a biomarker to monitor treatment response and neurodegeneration in progressive MS [24, 25]. Other studies have showed a reduction in NfL chain quantification after Siponimod or Ocrelizumab treatment in patients with SPMS or PMS respectively [26, 27].

#### Clinical assessments

At each timepoint patients will undergo a thorough neurological examination to estimate the patient’s disability progression, comparing pre- and post- treatment outcomes. Each central nervous system (CNS) functional domain will be addressed with the Expanded Disability Status Scale (EDSS). The Timed 25-foot Walk test (T25FW) and the Nine-Hole Peg Test (9HPT) will be performed as well.

EDSS is the main scale to evaluate the CNS functional domains. It is used to describe disease status at diagnosis and disease progression in MS patients; moreover, it is used to assess the effectiveness of therapeutic interventions in clinical trials [28]. It consists of an ordinal rating system ranging from 0 (normal neurological status) to 10 (death due to MS) in 0.5 increments interval (when reaching EDSS 1). The lower scale values of the EDSS measure impairments based on the neurological examination, while the upper range of the scale (> EDSS 6) measures handicaps of MS patients. The determination of EDSS 4–6 is heavily dependent on walking ability. In this trial we want to assess if there are any changes in EDSS that may suggest an effect of Forza™ on CNS function.

The T25FW and 9HPT tests are used to evaluate both upper and lower limb motor performance. The T25FW test is a quantitative measure of ambulation and has been used in clinical MS research for many years [29]. The 9HPT measures manual dexterity and gives a quantitative measure of arm and hand function. It is performed in both the dominant and nondominant hand and it has high interrater reliability [30].

#### Neuropsychological evaluations

Cognition will be assessed by symbol digit modalities test (SMDT) and Montreal Cognitive Assessment (MoCA).

The SMDT, part of the Brief International Cognitive Assessment for Multiple Sclerosis (BICAMS) battery [31], is the most commonly used neuropsychological test of processing speed in MS due to its ease of administration, reliability, and high sensitivity to cognitive impairment and cognitive changes in MS [32–36]. Moreover, it has been extensively studied due to its ability to appropriately mirror cognitive performance in its entirety [36]. It presents a symbol-digit pairing key at the top of the page and a series of symbols below. For 90 seconds, the patient orally indicates the matching digit to the random array of symbols [37].

The MoCA allows for the testing of a wider range of cognitive functions such as short-term memory, executive functions, visuospatial abilities, language, attention, concentration and working memory, and temporal-spatial orientation [38]. It has been shown to be a sensitive tool to detect cognitive impairment or cognitive changes in many diseases, including MS [39–41]. It has been added to the neuropsychological evaluation to allow for a deeper comprehension of the patient cognitive function and its potential change after the treatment.

#### Patient Reported Outcomes (PROs)

In addition to clinical and paraclinical measures, PROs, including MSWS-12, HADS and the OAB-q, will be collected. PROs provide a relevant measure of the impact of MS symptoms and in addition on the quality of life from the participants’ perspective [42], thus, they allow for the monitoring of Forza™ assumption effects on disease progression through the comparison of pre- and post-treatment scores.

MSWS-12 explores the impact of MS on walking and has shown strong correlation with T25FW test performance [43].

HADS is a rapid questionnaire developed to screen anxiety, depression, and limited concentration spans among patients in hospital settings [44] and it has been recently validated for the evaluation of the psychological burden of MS patients [45].

The OAB questionnaire (OAB-q) consists of an 8-item Symptom Bother scale and 25 health-related quality of life items that form four subscales (Coping, Concern, Sleep, and Social Interaction). This questionnaire examines bladder dysfunction symptoms, in particular how distressed MS patients are by frequent urination, urgency, nocturia and urge incontinence. Patients respond to each statement on a 6-point Likert scale: 1 (none of the time), 6 (all of the time) [46].

#### Brain magnetic resonance imaging

Brain MRI scans will be obtained using 3.0 Tesla scanners (Siemens). The following sequences will be collected with a standardized protocol of acquisition and careful guidelines for patients repositioning: axial T2 weighted Turbo Spin Echo (TSE); axial FLAIR; high resolution 3D sagittal T1-weighted sequence; axial DTI sequence (55 contiguous, 2.5 mm thick, slices, #DW direction = 64). To investigate the potential effect of Forza™ on brain metabolism (concentration of glutamate, N-acetylaspartate, creatine, choline) and microstructure, a subgroup of 25 patients (enrolled at the MS center of Genoa) will undergo MRI with a multi-shell diffusion-weighted (DWI) sequence and proton magnetic resonance spectroscopy (1H-MRI) in addition to the routine sequences. Movements will be minimized using foam padding and ear blocks. The locations of the 1H-MRI acquisitions will be determined from axial FLAIR, with the volume of interest (VOI) parallel to the anterior commissure-posterior commissure line going through the subcortical area.

The total duration of MRI acquisition will be approximately 50 min. From MRI images, new lesions occurrence, enlarging lesions and gadolinium-enhancing lesions will be collected.

#### Optical Coherence Tomography (OCT)

Change in retinal nerve fiber layer (RNFL) thickness (μm) and macular volume will be assessed for both eyes using OCT to evaluate axonal damage and disease progression.

Specifically, Spectral-Domain-OCT (Spectralis, Heidelberg-Engineering) imaging of eyes will be performed in a dark enclosed room. Scans will be processed by a certified neurologist, in accordance with the APOSTEL recommendations [47]. The following scans will be performed:

- Peripapillary RNFL (pRNFL) will be obtained with a 360° RNFL-B circle scan located at 3.4 cm from the center of the optic nerve head.
- Macular volumetric scans including 25 single horizontal axial B-scans will be acquired in a rectangular section centered over the macula. Macular scans will be segmented automatically into different layers using the Heidelberg Eye Explorer mapping software version 6.0.9.0. Macular layer volumes will be measured using the software’s segmentation algorithm.

Scans violating international-consensus quality-control criteria (OSCAR-IB) [48] will be excluded. The thickness of the global pRNFL and ganglion cell-inner plexiform layer (GCIPL) will be determined. The inter-eye differences of the pRNFL and GCIPL will be calculated. For each analysis we are going to use both the inter-eye percentage difference (IEPD, a dimensionless metric) and inter-eye absolute difference (IEAD, in μm) from the ETDRS grid. Retinal asymmetry will be defined based on a 4 μm cut-off for the GCIPL IEAD [49].

#### Neurophysiology assessment: Motor Evoked Potentials (MEPs)

Evoked potentials (EPs) are used to evaluate the impact of MS pathology on nervous function [50, 51]. In particular, MEPs have been shown to correlate with measures of motor performance [52], to improve under treatment [53, 54] and to predict disability worsening [55]. Therefore, MEPs may represent an ideal instrumental marker for monitoring motor disability in progressive MS [56].

In this study upper limbs MEPs will be evaluated. Electromyographic (EMG) activity will be recorded from the abductor pollicis brevis muscles of both sides using Ag±AgCl surface electrodes (0.9 cm diameter) placed over the muscle belly, with the reference over the metacarpophalangeal joint. Stimulation of the motor cortex will be performed with a Magstim 200 stimulator. A round coil (outer diameter 12 cm) will be centered over a point marked on the scalp at Cz (International 10±20 System) with the handle pointing posteriorly. Stimuli will be delivered on Cz from a coil point A and point B at a rate of one every 5 s.

### Data collection and data entry

All the clinical data will be collected and saved in a browser-based metadata-driven capture system, Research Electronic Data Capture (REDCap) by the personnel from the two centers. REDCap will be accessible to the Data Management and statistician staff where the data entry, data cleaning and quality control activities will be organized. Query will be raised if one or more data are unclear or contradictory.

### MedDRA dictionary

Medical events reported as Comorbidities and Complications will be classified using the last available version of the Medical Dictionary for Regulatory Activities (MedDRA), an international medical terminology dictionary applied by regulatory authorities and by pharmaceutical industries. The three levels of codes (SOC–PT–LLT) will be implemented into the database.

### Statistical analysis

A sample of 50 patients guarantees a 90% power to detect an absolute difference of 20% in the NfL percentage change (assuming SD = 40%) during the treatment period as compared to the pre-treatment period.

All safety and efficacy data will be analyzed using descriptive statistics. Safety data will be assessed by reporting and describing all the adverse events. Decisions following the safety pattern will be discussed by the study investigators, but no logic based on statistical test was set. NfL data will be summarized by the geometric mean. Continuous variables will be described with the number of patients with valid observations, mean, standard deviation, median, minimum, and maximum value. If necessary, additional descriptive statistics may be calculated (in particular, for non-normally distributed data, the median with interquartile range). Categorical data will be described by frequencies and related percentages within variables. A p-value of 0.05 will be used as the cut off for statistical significance (no statistical test will be used for safety data).

Any comparisons between pre- and post- changes in continuous variables will be evaluated with paired sample t-test or Wilcoxon signed rank-test, as appropriate.

Depending on the results of interest, a stratified or an exploratory analysis may be conducted.

### Ethical aspects and study status

All participating centers have obtained required ethics approval by the local Ethic Committees (EC). This study was approved by the Regional Ethic Committee of Liguria (CER Liguria) with id 12042.

Following the approval of the Ethic Committees (EC), this study is now being conducted in accordance with the study protocol, the current version of the Declaration of Helsinki, applicable Good Clinical Practices (GCP) guidelines and with the specific Italian regulations on interventional studies.

## Discussion

Effective treatments to limit disability accrual in progressive MS patients represent an unmet need in current MS treatment. Due to their favorable benefit-risk balance, nutraceutical interventions might benefit as add-on treatment in MS.

Many data support the effect of osmotin and its mammalian homologue adiponectin in promoting anti-inflammatory response and neuroprotection [12, 14–17]. Forza™ consists of lyophilized leaves of Actinidia Deliciosa plants which are bioengineered to produce a high quantity of recombinant osmotin and it represents the ideal way to deliver this protein [20]. This study will be the first to explore safety and efficacy of an osmotin-based nutraceutical as an adjuvant treatment in real clinical practice.

PMS has been chosen as target of this trial because the low impact of current therapies in PMS are unlikely to overshadow possible effects of this new nutraceutical product, something that is otherwise likely to happen with high efficacy treatments used in RRMS.

Since this is the first study using Forza™, tolerability and safety issues will be monitored closely to verify its safety, the primary outcome of this study. In this regard patient will be evaluated at each time point and they will be encouraged to notify the medical personnel every positive or negative effect that may be attributable to Forza™.

NfL chain quantification has been chosen as the coprimary outcome since it serves as a biomarker to monitor treatment response and neurodegeneration in MS [25, 26]. The evaluation if this nutraceutical product’s ability to lower NfL serum concentrations, as shown by other treatments in SPMS and PMS [26, 27] would be of great interest. Clinical, neurophysiological, and imaging biomarkers of disease progression and disability accrual in different domains will be assessed as secondary endpoint to perceive any potential beneficial effects of Forza™ as an add-on therapy in PMS.

As an exploratory analysis, a subgroup of 25 patients in the Genoa SM center will undergo DWI and 1H MRI in addition to routine sequences to investigate the potential effect of Forza™ on brain metabolism (concentration of glutamate, N-acetyl aspartate, creatine, choline) and microstructure. Specifically changes in brain metabolites indicating remyelination will be monitored.

One of the strengths of this study is the comprehensive approach, as many efficacy outcome measures are investigated to define disease status from 6 months before treatment exposure, combining them with patient-reported measures of symptom burden.

This study has several limitations. One of these is the single arm, baseline vs treatment design that precludes drawing firm conclusions on efficacy. The main issue of a baseline vs treatment design is a potential regression-to-the-mean phenomenon. Patients who are in a particularly severe disease status and are enrolled in a study, naturally tend to improve over time. A second limitation is the small sample size, as it may be not insufficient to fully explore Forza™ safety. However, these preliminary results could inspire further larger investigations. Moreover 50 patients guarantee a 90% power to detect a reduction of 20% of Neurofilament change rate (assuming SD = 40%) during the treatment period as compared to the pre-treatment period, allowing for the assessment of the potential efficacy of this new nutraceutical product.

In conclusion, this study is expected to provide preliminary evidence for the use of osmotin protein, specifically in the form of Forza™ capsules or powder by patients with progressive multiple sclerosis after a 6 months course. Given the known limitations of an uncontrolled study, the small sample, the limited intervention period, further trials will be needed.

## Supporting information

S1 Fig SPIRIT schedule of enrollment, intervention, and assessments. (DOCX)

S1 File SPIRIT checklist 2013. (PDF)

S2 File WHO trial registration data set. (DOCX)

S3 File Study protocol. (DOCX)
