# Supplementary material for: Study protocol: Exploratory trial of Forza™, an osmotin-based nutraceutical as adjuvant for the treatment of progressive multiple sclerosis
Source: PLoS One. 2025 Feb 27;20(2):e0311214. doi: 10.1371/journal.pone.0311214 (PMC11867331; doi:10.1371/journal.pone.0311214)
Supplement: S1 Fig — (DOCX) [file pone.0311214.s001.docx]

| FIGURE 1. SPIRIT Schedule of EnrolLment, intervenTion and assessments | | | | |
| --- | --- | --- | --- | --- |
|  | **Pre-treatment**  **-6 months** | **Pre-treatment**  **0 month** | **Follow up**  **+1 month** | **Follow up**  **+6 months** |
|  | **(-6M)** | **(0M)** | **(M1)** | **(M6)** |
| *Demography* | 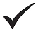 |  |  |  |
| *MS diagnosis and history* | 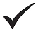 |  |  |  |
| *Clinical evaluation*  *(any changes in therapy, any relapses)* |  | 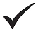 | 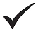 | 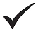 |
| *Eligibility screen* | 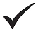 |  |  |  |
| *Informed consent signing* | 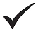 |  |  |  |
| *Treatment assumption beginning* |  | 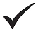* |  |  |
| *NfL* | 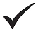 | 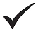 | 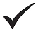 | 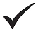 |
| *MEPs* | 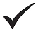 | 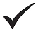 |  | 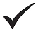 |
| *OCT* | 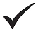 | 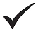 | 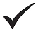 | 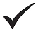 |
| *EDSS* | 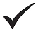 | 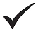 | 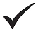 | 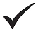 |
| *T25FW* | 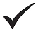 | 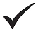 | 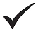 | 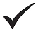 |
| *MSWS12* | 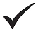 | 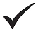 | 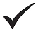 | 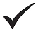 |
| *9HPT* | 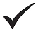 | 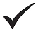 | 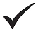 | 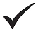 |
| *MOCA* | 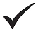 | 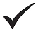 | 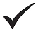 | 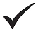 |
| *SDMT* | 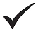 | 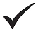 | 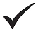 | 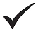 |
| *HADS* | 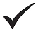 | 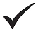 | 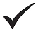 | 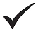 |
| *OAB* | 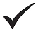 | 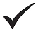 | 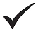 | 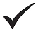 |
| *Brain MRI* | 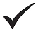 | 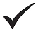 | 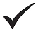 | 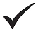 |
| *MRI with DWI and 1H-MRI*** | 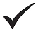 | 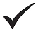 | 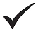 | 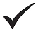 |
| *Treatment compliance* |  |  | 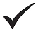 | 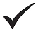 |
| *AE collection* |  |  | 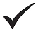 | 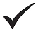 |
| ** Only after the execution of all assessments*  ***Only for a subgroup of patients* | | | | |
