# Supplementary material for: Study protocol: Exploratory trial of Forza™, an osmotin-based nutraceutical as adjuvant for the treatment of progressive multiple sclerosis
Source: PLoS One. 2025 Feb 27;20(2):e0311214. doi: 10.1371/journal.pone.0311214 (PMC11867331; doi:10.1371/journal.pone.0311214)
Supplement: S2 File — (DOCX) [file pone.0311214.s003.docx]

**WHO Trial Registration Data Set**

**1) Primary Registry and Trial Identifying Number**

**Primary registry: ClinicalTrials.gov**

**ID number**: NCT05937802

**2) Date of Registration in Primary Registry**
10/07/2023.

**3) Secondary Identifying Numbers**
Other study ID number: 12042.

**4) Source(s) of Monetary or Material Support**
FORZA™️ supplied by 9th Dimension Biotech, Inc, United States of America.

**5) Primary Sponsor**
Ospedale Policlinico San Martino.

**6) Secondary Sponsor(s)**

Not applicable.

**7)** **Contact for Public Queries**
Mail: [m.inglese@unige.it](mailto:m.inglese@unige.it). Telephone Number: 0105557290. Postal Address: Largo Daneo 3, Genova, 16132.

**8) Contact for Scientific Queries**

Mail: [m.inglese@unige.it](mailto:m.inglese@unige.it). Telephone Number: 0105557290. Postal Address: Largo Daneo 3, Genova, 16132. Affiliations: Department of Neurosciences, Rehabilitation, Ophthalmology, Genetics, Maternal and Child Health (DiNOGMI), University of Genoa, Genoa, Italy; IRCCS Ospedale Policlinico San Martino, UOC Clinica Neurologica, Genoa, Italy.

Mail: [centrosm@unige.it](mailto:centrosm@unige.it). Telephone Number: 0105557290. Postal Address: Largo Daneo 3, Genova, 16132. Affiliations: Department of Neurosciences, Rehabilitation, Ophthalmology, Genetics, Maternal and Child Health (DiNOGMI), University of Genoa, Genoa, Italy.

**9) Public Title**
Osmotin Plant Protein for Progressive Multiple Sclerosis.

**10) Scientific Title**
Exploratory trial of Forza, a novel nutraceutical from Actinidia Deliciosa plants bioengineered to bioencapsulate the osmotin plant protein as adjuvant for the treatment of progressive multiple sclerosis.

**11) Countries of Recruitment**
Italy.

**12) Health Condition(s) or Problem(s) Studied**
Multiple Sclerosis.

**13) Intervention(s)**

Dietary Supplement: Osmotin through FORZA™️, a novel nutraceutical from Actinidia Deliciosa Plants bioengineered to Bio-encapsulate the Osmotin Plant Protein.

The treatment consists in the oral administration of a dosage of 5 grams per day of Forza™️, provided in the form of capsules or powder (9th Dimension Biotech, Inc.). Each capsule contains approximately 0.7 grams of Forza™️. The capsules will be administered as follows: 4 capsules in the morning and 3 capsules in the evening for 6 months.

**14) Key Inclusion and Exclusion Criteria**
Inclusion Criteria: signed written informed consent, diagnosis of progressive multiple sclerosis (PMS), Expanded Disability Status Scale EDSS ≤ 6.5.

Exclusion Criteria: contraindications to MRI, pregnancy, HIV positivity, severe renal, hepatic, oncological, hematological and psychiatric diseases.

**15) Study Type**

This is a prospective, multicenter, single-arm interventional, baseline vs treatment study.

**16) Date of First Enrollment**
January 2023.

**17) Sample Size**
Number of participants that the trial plans to enroll in total: 50.

Number of participants that the trial has enrolled: 25/50.

**18) Recruitment Status**
Recruitment status of this trial: recruiting.

**19) Primary Outcome(s)**
Incidence and severity of treatment-related adverse events after 1 month of therap. Time Frame: 1 month (after 1 month of treatment).

Incidence and severity of treatment-related adverse events after 6 months of therapy. Time Frame: 6 months (after 6 months of treatment).

**20) Key Secondary Outcomes**

Change in Expanded Disability Status Scale (EDSS). Time frame: 12 months (6 months before starting treatment, at baseline and both after one month and six months of treatment).

Change in Timed 25 Foot Walk (T25FW). Time Frame: 12 months (6 months before starting treatment, at baseline and both after one month and six months of treatment).

Change in 12-item Multiple Sclerosis Walking Scale (MSWS12). Time Frame: 12 months (6 months before starting treatment, at baseline and both after one month and six months of treatment).

Change in Nine-Hole Peg Test (9HPT). Time Frame: 12 months (6 months before starting treatment, at baseline and both after one month and six months of treatment).

Change in Montreal Cognitive Assessment (MOCA). Time Frame: 12 months (6 months before starting treatment, at baseline and both after one month and six months of treatment).

Change in Symbol Digit Modalities Test (SDMT). Time Frame: 12 months (6 months before starting treatment, at baseline and both after one month and six months of treatment).

Change in patient self-evaluation of depression and anxiety recorded with Hospital Anxiety Depression Scale (HADS). Time Frame: 12 months (6 months before starting treatment, at baseline and both after one month and six months of treatment).

Change in bladder domain function recorded with Overactive Bladder (OAB) questionnaire. Time Frame: 12 months (6 months before starting treatment, at baseline and both after one month and six months of treatment).

The impact of Forza™️ on neurophysiology in PMS. Time Frame: 12 months (6 months before starting treatment, at baseline and both after one month and six months of treatment).

The impact of Forza™️ on retinal atrophy in PMS. Time Frame: 12 months (6 months before starting treatment, at baseline and both after one month and six months of treatment).

Change in serum neurofilament Light Chain (NfL) levels to verify the neuroprotective action of Forza™️ in PMS. Time Frame: 12 months (6 months before starting treatment, at baseline and both after one month and six months of treatment).

Change in brain metabolism as concentration of glutamate, N-acetylaspartate, creatine and choline. Time Frame: 12 months (6 months before starting treatment, at baseline and both after one month and six months of treatment).

Change in brain microstructure. Time Frame: 12 months (6 months before starting treatment, at baseline and both after one month and six months of treatment).

**21) Ethics Review**
All participating centers have obtained required ethics approval by the local Ethic Committees (EC). This study was approved by the Regional Ethic Committee of Liguria (CER Liguria) with id 12042.

Following the approval of the Ethic Committees (EC), this study is now being conducted in accordance with the study protocol, the current version of the Declaration of Helsinki, applicable Good Clinical Practices (GCP) guidelines and with the specific Italian regulations on interventional studies.

**22) Completion date**
The study is ongoing.

**23) Summary Results**
The study is ongoing, no results have been produced yet.

**24) IPD sharing statement**

Upon completion of data collection and statistical analysis, a final report, including a review of the methods and objectives, together with presentation and discussion of results, will be drawn. Results of the study will be presented at national and international conferences.
