# Supplementary material for: Study protocol: Exploratory trial of Forza™, an osmotin-based nutraceutical as adjuvant for the treatment of progressive multiple sclerosis
Source: PLoS One. 2025 Feb 27;20(2):e0311214. doi: 10.1371/journal.pone.0311214 (PMC11867331; doi:10.1371/journal.pone.0311214)
Supplement: S3 File — (DOCX) [file pone.0311214.s004.docx]

| **CLINICAL STUDY PROTOCOL** | |
| --- | --- |
| **Exploratory trial of Forza, a novel nutraceutical from Actinidia Deliciosa plants bioengineered to bioencapsulate the osmotin plant protein as adjuvant for the treatment of progressive multiple sclerosis** | |
|  | |
| **STUDY CODE:** | **Forza trial** |
| **VERSION AND DATE:** | Version Number: 03 – October 20^th^, 2021 |
|  | |

| **SYNOPSIS** | |
| --- | --- |
|  | |
| **Title** | Exploratory trial of Forza, a novel nutraceutical from Actinidia Deliciosa plants bioengineered to bioencapsulate the osmotin plant protein as adjuvant for the treatment of progressive multiple sclerosis |
|  |  |
| **Protocol code** | Forza trial |
|  |  |
| **Version** | Version Number: 03 – October 20^th^, 2021 |
|  |  |
| **Coordinator** | Matilde Inglese |
|  |  |
| **Sites** | The recruitment will be carried out by Neurologists at the MS Centres of San Martino Hospital in Genoa and of the Sant’Andrea University Hospital,Rome. |
|  |  |
| **Background and rationale for the study** | Multiple sclerosis (MS) is the first cause of progressive neurological disability in young adults.  In the relapsing-remitting (RR) form of the disease, the pathophysiology is dominated by the inflammatory response, and a range of effective immune-modulating therapies have been successfully developed. In progressive MS (PMS), different pathophysiologic mechanisms seem to interact, resulting in myelin damage and neurodegeneration through incompletely understood events. Therefore, while targeting inflammatory pathways has advanced the efficacy of treatments in RRMS, progressive forms lag behind^1^. Furthermore, progression of disability develops in MS independently of disease phase: disability can accrue insidiously also during the RR phase of the disease^2,3^. Furthermore, a significant percentage of people with RRMS, even though treated with the most effective therapies, still develop secondary progressive MS (SPMS)^4^.  Osmotin is a protein initially characterized as associated with stress adaptation in plant cells^5^. Actinidia Deliciosa plants overexpressing the osmotin protein were developed at the University of Tuscia in Italy, and tested for ten years for their improved agronomic and nutritional qualities^6^. In addition, it has been shown in different plant species that the overexpression of the osmotin plant gene increases the production of flavon-3-ols and other anti-inflammatory plant compounds ^30^. In mammals, osmotin binds the adiponectin receptor due to the 3D structure homology between the two proteins^7^. As such, it may share some of the anti-inflammatory effects of adiponectin^8-10, 28^. Indeed, this appears to be the case with the attenuation of LPS-induced neuroinflammation^11^ and with preventive effects on vascular inflammation in atherosclerosis^10^. Osmotin also protects against glutamate-induced synaptic dysfunction and neurodegeneration in the rat brain, and attenuates LPS-induced neuroinflammation^11^. However, adiponectin also has pro-inflammatory functions^12^. High and low molecular weight isoforms of the adiponectin molecule may play different roles in inflammation^13,14^. This may explain conflicting results on the role of adiponectin in MS^15-25^. However, because osmotin and adiponectin have different amino acid sequences, with the exception of the active site ^29^, osmotin may not manifest the negative effects that may be associated with adiponectin polymers. Additionally, the anti-inflammatory effects of Actinidia plants are well-documented ^27^.  Based on these premises, a nutraceutical product (commercial name Forza) has been developed by 9^th^ Dimension Biotech, Inc., consisting of the lyophilized leaves of the A. Deliciosa plants bioencapsulating recombinant osmotin, with the goal of offering the combined benefits of the osmotin protein in conjunction with the enhanced anti-inflammatory properties of the bioengineered A. Deliciosa plants to consumers. In fact, as recently as in 2016, it has been shown that the bioencapsulation of protein drugs in lyophilized plant cells represents an ideal method of oral protein drug delivery to patients, which surpasses all other methods of protein drug delivery both in safety and efficacy^26^. Therefore, it may be worth exploring the effects of Forza in MS, where Forza may play both anti-inflammatory and neuroprotective effects. Considering the strong need to identify therapies for the progressive forms of the disease, we here propose to explore the effects of Forza in progressive MS (PMS). Moreover, the low impact of current therapies in PMS may not overshadow possible effects of Forza, something that would probably happen with high efficacy treatments in RRMS. |
|  |  |
| **Objectives** | This study aims at verifying the safety and to explore the clinical and biological efficacy of Forza in PMS. |
|  |  |
| **Design** | Prospective, multicenter, single-arm non-interventional study. |
|  |  |
| **Patients** | Patients with a progressive form of MS will be enrolled by the MS Center of the Clinica Neurologica San Martino Hospital in Genoa and of Sant ’Andrea Hospital of Rome.  A sample of 50 patients guarantee a 90% power to detect a reduction of 20% in the Neurofilament change rate (assuming a SD=40%) during the treatment period as compared to the pre-treatment period. |
|  |  |
| **Inclusion/exclusion criteria** | patients with PMS meeting the inclusion criteria will be included in the trial. |
|  |  |
| **Methods** | Patients meeting all inclusion/exclusion criteria will be included in the study after having provided their written informed consent.  They will undergo two pre-treatment assessments: at -6 months (-6M) and at baseline visit (M0) and two post-treatment assessments at month 1 (M1), and at month 6 (M6).  The treatment will consist in the oral administration of a minimum dosage of 5 grams per day of Forza, provided in the form of capsules. Each capsule will contain approximately 0.7g of Forza. The capsules will be administered as follow: 4 capsules in the morning and 3 additional capsules in the evening from baseline () to month 6 (M6).  At each timepoint the following assessments will be evaluated: electrophysiological (motor evoked potentials – MEPs; somatosensory evoked potentials – SEPs; visual evoked potentials – VEPs), optical coherence tomography (OCT), and neurofilaments (NfL).  In addition, clinician assessed outcomes (CAO), patient reported outcomes (PRO) and performance measure (PF) will be acquired for the motor domain (Expanded Disability Status Scale – EDSS; Timed 25 Foot Walk - T25FW; 12-item Multiple Sclerosis Walking Scale - MSWS12; Nine-Hole Peg Test - 9HPT), for the cognitive domain (Montreal Cognitive Assessment - MOCA; Symbol Digit Modalities Test - SDMT), for the emotional domain (Hospital Anxiety and Depression Scale - HADS), and for the bladder domain (Overactive Bladder - OAB).  Brain MRI will also be obtained at each timepoint as standard of care procedure.  In order to investigate the potential effect of Forza on brain metabolism (concentration of glutamate, N-acetylaspartate, creatine, choline) and microstructure, a subgroup of 25 patients (enrolled at the University of Genoa) will undergo MRI with a multi-shell diffusion-weighted (DWI) sequence and proton magnetic resonance spectroscopy (1H-MRI) in addition to routine sequences. |
| **Endpoints** | The primary outcome of the study will be safety, and the co-primary efficacy outcome will be a reduction in the NfL change over 1 and 6 months as compared to the pre-treatment NfL change. |
| **Statistical analysis** | The NfL percentage changes will be compared pre and post treatment within patients by a non-parametric paired Wilcoxon test. |
| **Expected results** | We expect to verify the safety of an add-on therapy with *Forza* in PMS and to obtain clinical, paraclinical and laboratory information on the effects of *Forza* in this condition. |
|  |  |
| **References** | 1. Dangond F, Donnelly A, Hohlfeld R, Lubetzki C, Kohlhaas S, Leocani L, Ciccarelli O, Stankoff B, Sormani MP, Chataway J, Bozzoli F, Cucca F, Melton L, Coetzee T, Salvetti M. Facing the urgency of therapies for progressive MS - a Progressive MS Alliance proposal. Nat Rev Neurol. 2021 Mar;17(3):185-192. doi: 10.1038/s41582-020-00446-9. Epub 2021 Jan 22. PMID: 33483719. 2. University of California, San Francisco MS-EPIC Team, Cree BAC, Hollenbach JA, Bove R, Kirkish G, Sacco S, Caverzasi E, Bischof A, Gundel T, Zhu AH, Papinutto N, Stern WA, Bevan C, Romeo A, Goodin DS, Gelfand JM, Graves J, Green AJ, Wilson MR, Zamvil SS, Zhao C, Gomez R, Ragan NR, Rush GQ, Barba P, Santaniello A, Baranzini SE, Oksenberg JR, Henry RG, Hauser SL. Silent progression in disease activity-free relapsing multiple sclerosis. Ann Neurol. 2019 May;85(5):653-666. doi: 10.1002/ana.25463. Epub 2019 Mar 30. PMID: 30851128; PMCID: PMC6518998. 3. Kappos L, Wolinsky JS, Giovannoni G, Arnold DL, Wang Q, Bernasconi C, Model F, Koendgen H, Manfrini M, Belachew S, Hauser SL. Contribution of Relapse-Independent Progression vs Relapse-Associated Worsening to Overall Confirmed Disability Accumulation in Typical Relapsing Multiple Sclerosis in a Pooled Analysis of 2 Randomized Clinical Trials. JAMA Neurol. 2020 Sep 1;77(9):1132-1140. doi: 10.1001/jamaneurol.2020.1568. PMID: 32511687; PMCID: PMC7281382. 4. Green AJ. Potential Benefits of Early Aggressive Treatment in Multiple Sclerosis. JAMA Neurol. 2019 Mar 1;76(3):254-256. doi: 10.1001/jamaneurol.2018.4932. PMID: 30644966. 5. Singh NK, Bracker CA, Hasegawa PM, Handa AK, Buckel S, Hermodson MA, Pfankoch E, Regnier FE, Bressan RA. Characterization of osmotin : a thaumatin-like protein associated with osmotic adaptation in plant cells. Plant Physiol. 1987 Oct;85(2):529-36. doi: 10.1104/pp.85.2.529. PMID: 16665731; PMCID: PMC1054289. 6. Rugini, E., Cristofori, V., Martignoni, D., Gutierrez-Pesce, P., Orlandi, S., Brunori, E., Biasi, R., Muleo, R. and Magro, P. (2011). KIWIFRUIT TRANSGENICS FOR OSMOTIN GENE AND INOCULATION TESTS WITH BOTRYTIS CINEREA AND CADOPHORA LUTEO-OLIVACEA. Acta Hortic. 913, 197-203 DOI: 10.17660/ActaHortic.2011.913.25 7. Narasimhan ML, Coca MA, Jin J, Yamauchi T, Ito Y, Kadowaki T, Kim KK, Pardo JM, Damsz B, Hasegawa PM, Yun DJ, Bressan RA. Osmotin is a homolog of mammalian adiponectin and controls apoptosis in yeast through a homolog of mammalian adiponectin receptor. Mol Cell. 2005 Jan 21;17(2):171-80. doi: 10.1016/j.molcel.2004.11.050. Erratum in: Mol Cell. 2005 Feb 18;17(4):611. PMID: 15664187. 8. Carbone F, La Rocca C, Matarese G. Immunological functions of leptin and adiponectin. Biochimie. 2012 Oct;94(10):2082-8. doi: 10.1016/j.biochi.2012.05.018. Epub 2012 Jun 26. PMID: 22750129. 9. Takahashi Y, Watanabe R, Sato Y, Ozawa N, Kojima M, Watanabe-Kominato K, Shirai R, Sato K, Hirano T, Watanabe T. Novel phytopeptide osmotin mimics preventive effects of adiponectin on vascular inflammation and atherosclerosis. Metabolism. 2018 Jun;83:128-138. doi: 10.1016/j.metabol.2018.01.010. Epub 2018 Feb 2. PMID: 29410350. 10. Liu J, Sui H, Zhao J, Wang Y. Osmotin Protects H9c2 Cells from Simulated Ischemia-Reperfusion Injury through AdipoR1/PI3K/AKT Signaling Pathway. Front Physiol. 2017 Sep 25;8:611. doi: 10.3389/fphys.2017.00611. PMID: 28993734; PMCID: PMC5622187. 11. Badshah H, Ali T, Kim MO. Osmotin attenuates LPS-induced neuroinflammation and memory impairments via the TLR4/NFκB signaling pathway. Sci Rep. 2016 Apr 20;6:24493. doi: 10.1038/srep24493. PMID: 27093924; PMCID: PMC4837357. 12. Fantuzzi, G. Adiponectin and inflammation: Consensus and controversy. J Allergy Clin Immunol 2008; 121(2): 326–330. 13. Neumeier M, Weigert J, Schäffler A, Wehrwein G, Müller-Ladner U, Schölmerich J, Wrede C, Buechler C. Different effects of adiponectin isoforms in human monocytic cells. J Leukoc Biol. 2006 Apr;79(4):803-8. doi: 10.1189/jlb.0905521. Epub 2006 Jan 24. PMID: 16434692. 14. Song H, Chan J, Rovin BH. Induction of chemokine expression by adiponectin in vitro is isoform dependent. Transl Res. 2009 Jul;154(1):18-26. doi: 10.1016/j.trsl.2009.04.003. Epub 2009 May 9. PMID: 19524870; PMCID: PMC2727280. 15. Piccio L, Stark JL, Cross AH. Chronic calorie restriction attenuates experimental autoimmune encephalomyelitis. J Leukoc Biol. 2008 Oct;84(4):940-8. doi: 10.1189/jlb.0208133. Epub 2008 Aug 4. PMID: 18678605; PMCID: PMC2638732. 16. Hietaharju A, Kuusisto H, Nieminen R, Vuolteenaho K, Elovaara I, Moilanen E. Elevated cerebrospinal fluid adiponectin and adipsin levels in patients with multiple sclerosis: a Finnish co-twin study. Eur J Neurol. 2010 Feb;17(2):332-4. doi: 10.1111/j.1468-1331.2009.02701.x. Epub 2009 Jun 15. PMID: 19538214. 17. Piccio L, Cantoni C, Henderson JG, Hawiger D, Ramsbottom M, Mikesell R, Ryu J, Hsieh CS, Cremasco V, Haynes W, Dong LQ, Chan L, Galimberti D, Cross AH. Lack of adiponectin leads to increased lymphocyte activation and increased disease severity in a mouse model of multiple sclerosis. Eur J Immunol. 2013 Aug;43(8):2089-100. doi: 10.1002/eji.201242836. Epub 2013 Jun 7. PMID: 23640763; PMCID: PMC3901539. 18. Devorak J, Mokry LE, Morris JA, Forgetta V, Davey Smith G, Sawcer S, Richards JB. Large differences in adiponectin levels have no clear effect on multiple sclerosis risk: A Mendelian randomization study. Mult Scler. 2017 Oct;23(11):1461-1468. doi: 10.1177/1352458516681196. Epub 2016 Dec 7. PMID: 27903934. 19. Çoban A, Düzel B, Tüzün E, Tamam Y. Investigation of the prognostic value of adipokines in multiple sclerosis. Mult Scler Relat Disord. 2017 Jul;15:11-14. doi: 10.1016/j.msard.2017.04.006. Epub 2017 Apr 20. PMID: 28641765. 20. Kvistad SS, Myhr KM, Holmøy T, Benth JŠ, Wergeland S, Beiske AG, Bjerve KS, Hovdal H, Midgard R, Sagen JV, Torkildsen Ø. Serum levels of leptin and adiponectin are not associated with disease activity or treatment response in multiple sclerosis. J Neuroimmunol. 2018 Oct 15;323:73-77. doi: 10.1016/j.jneuroim.2018.07.011. Epub 2018 Jul 24. PMID: 30196837. 21. Signoriello E, Lus G, Polito R, Casertano S, Scudiero O, Coletta M, Monaco ML, Rossi F, Nigro E, Daniele A. Adiponectin profile at baseline is correlated to progression and severity of multiple sclerosis. Eur J Neurol. 2019 Feb;26(2):348-355. doi: 10.1111/ene.13822. Epub 2018 Nov 27. PMID: 30300462. 22. Keyhanian K, Saxena S, Gombolay G, Healy BC, Misra M, Chitnis T. Adipokines are associated with pediatric multiple sclerosis risk and course. Mult Scler Relat Disord. 2019 Nov;36:101384. doi: 10.1016/j.msard.2019.101384. Epub 2019 Sep 5. PMID: 31550559. 23. Signoriello E, Mallardo M, Nigro E, Polito R, Casertano S, Di Pietro A, Coletta M, Monaco ML, Rossi F, Lus G, Daniele A. Adiponectin in Cerebrospinal Fluid from Patients Affected by Multiple Sclerosis Is Correlated with the Progression and Severity of Disease. Mol Neurobiol. 2021 Jun;58(6):2663-2670. doi: 10.1007/s12035-021-02287-z. Epub 2021 Jan 23. Erratum in: Mol Neurobiol. 2021 Feb 18;: PMID: 33486671. 24. Nyirenda MH, Fadda G, Healy LM, Mexhitaj I, Poliquin-Lasnier L, Hanwell H, Saveriano AW, Rozenberg A, Li R, Moore CS, Belabani C, Johnson T, O'Mahony J, Arnold DL, Yeh EA, Marrie RA, Dunn S, Banwell B, Bar-Or A. Pro-inflammatory adiponectin in pediatric-onset multiple sclerosis. Mult Scler. 2021 Feb 1:1352458521989090. doi: 10.1177/1352458521989090. Epub ahead of print. PMID: 33522403. 25. Kwon KC, Daniell H. Oral Delivery of Protein Drugs Bioencapsulated in Plant Cells. Mol Ther. 2016 Aug;24(8):1342-50. doi: 10.1038/mt.2016.115. Epub 2016 Jun 6. PMID: 27378236; PMCID: PMC5023392. 26. Harroud A, Manousaki D, Butler-Laporte G, Mitchell RE, Davey Smith G, Richards JB, Baranzini SE. The relative contributions of obesity, vitamin D, leptin, and adiponectin to multiple sclerosis risk: A Mendelian randomization mediation analysis. Mult Scler. 2021 Feb 19:1352458521995484. doi: 10.1177/1352458521995484. Epub ahead of print. PMID: 33605807. 27. Kim HK, Bae MJ, Lim S, Lee W, Kim S. A Water-Soluble Extract from *Actinidia arguta* Ameliorates Psoriasis-Like Skin Inflammation in Mice by Inhibition of Neutrophil Infiltration. Nutrients. 2018 Oct 2;10(10):1399. doi: 10.3390/nu10101399. PMID: 30279326; PMCID: PMC6213123. 28. Parida S, Siddharth S, Sharma D. Adiponectin, Obesity, and Cancer: Clash of the Bigwigs in Health and Disease. Int J Mol Sci. 2019 May 22;20(10):2519. doi: 10.3390/ijms20102519. PMID: 31121868; PMCID: PMC6566909. 29. Miele M, Costantini S, Colonna G (2011) Correction: Structural and Functional Similarities between Osmotin from *Nicotiana Tabacum* Seeds and Human Adiponectin. PLoS ONE 6(2): 10.1371/annotation/69357261-7e31-40e0-96ff-13cdc783c768. 30. Bhattacharya A, Saini U, Joshi R, Kaur D, Pal AK, Kumar N, Gulati A, Mohanpuria P, Yadav SK, Kumar S, Ahuja PS. Osmotin-expressing transgenic tea plants have improved stress tolerance and are of higher quality. Transgenic Res. 2014 Apr;23(2):211-23. doi: 10.1007/s11248-013-9740-5. Epub 2013 Aug 27. PMID: 23982743. |

**TABLE OF CONTENT**

FLOW CHART 9

INTRODUCTION 11

ETHICAL ASPECTS 13

STUDY OBJECTIVES 14

METHODS 15

STUDY TREATMENT 17

STUDY POPULATION 18

DATA MANAGEMENT AND STATISTICAL METHODS 20

PLAN FOR DISSEMINATION OF STUDY RESULTS 21

REFERENCES 22

| FORZA – Study flow chart | | | | |
| --- | --- | --- | --- | --- |
|  | **Pre-treatment**  **-6 months** | **Pre-treatment**  **0 month** | **Follow up**  **+1 month** | **Follow up**  **+6 months** |
|  | **(-6M)** | **(0M)** | **(1M)** | **(6M)** |
| *Demography* | 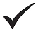 |  |  |  |
| *MS diagnosis and history* | 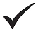 |  |  |  |
| *Clinical evaluation*  *(any changes in therapy, any relapses)* |  | 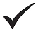 | 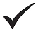 | 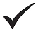 |
| *NfL* | 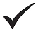 | 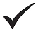 | 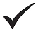 | 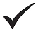 |
| *MEPs* | 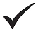 | 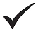 |  | 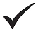 |
| *OCT* | 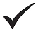 | 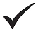 | 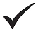 | 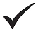 |
| *EDSS* | 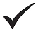 | 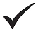 | 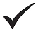 | 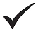 |
| *T25FW* | 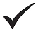 | 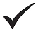 | 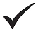 | 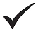 |
| *MSWS12* | 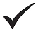 | 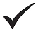 | 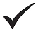 | 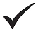 |
| *9HPT* | 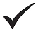 | 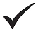 | 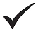 | 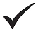 |
| *MOCA* | 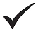 | 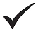 | 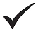 | 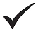 |
| *SDMT* | 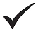 | 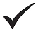 | 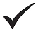 | 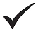 |
| *HADS* | 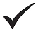 | 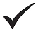 | 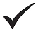 | 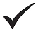 |
| *OAB* | 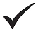 | 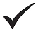 | 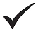 | 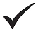 |
| *Brain MRI* | 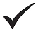 | 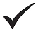 | 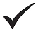 | 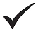 |
| *MRI with DWI and 1H-MRI*** | 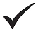 | 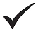 | 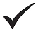 | 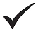 |
| *Treatment assumption* |  | 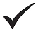* |  |  |
| *Treatment compliance* |  |  | 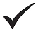 | 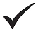 |
| *AE collection* |  |  | 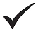 | 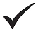 |
| ** Only after the execution of all assessments*  ***Only for a subgroup of patients* | | | | |

|  |  |
| --- | --- |
|  |  |
|  |  |
|  |  |
|  |  |

## INTRODUCTION

Background

Multiple sclerosis (MS) is the first cause of progressive neurological disability in young adults.

In the relapsing-remitting (RR) form of the disease, the pathophysiology is dominated by the inflammatory response, and a range of effective immune-modulating therapies have been successfully developed. In progressive MS (PMS), different pathophysiologic mechanisms seem to interact, resulting in myelin damage and neurodegeneration through incompletely understood events. Therefore, while targeting inflammatory pathways has advanced the efficacy of treatments in RRMS, progressive forms lag behind.^1^ Furthermore, progression of disability develops in MS independently of disease phase: disability can accrue insidiously also during the RR phase of the disease^2,3^. Furthermore, a significant percentage of people with RRMS, even though treated with the most effective therapies, still develop secondary progressive MS (SPMS)^4^.

**Rationale for the study**

Osmotin is a protein initially characterized as associated with stress adaptation in plant cells^5^. Actinidia Deliciosa plants overexpressing the osmotin protein were developed at the University of Tuscia in Italy, and tested for ten years for their improved agronomic and nutritional qualities^6^. In addition, it has been shown in different plant species that the overexpression of the osmotin plant gene increases the production of flavon-3ols and other anti-inflammatory plant compounds^30^. In mammals, osmotin binds the adiponectin receptor due to the 3D structure homology between the two proteins^7^. As such, it may share some of the anti-inflammatory effects of adiponectin^8-10, 28^. Indeed, this appears to be the case with the attenuation of LPS-induced neuroinflammation^11^ and with preventive effects on vascular inflammation in atherosclerosis^10^. Osmotin also protects against glutamate-induced synaptic dysfunction and neurodegeneration in the rat brain, and attenuates LPS-induced neuroinflammation^11^. However, adiponectin also has pro-inflammatory functions^12^. High and low molecular weight isoforms of the adiponectin molecule may play different roles in inflammation^13,14^. This may explain conflicting results on the role of adiponectin in MS^15-25^. However, because osmotin and adiponectin have different amino acid sequences, with the exception of the active site^29^, osmotin may not manifest the negative effects that may be associated with adiponectin polymers. Additionally, the anti-inflammatory effects of Actinidia plants are well-documented^27^.

Based on these premises, a nutraceutical product (commercial name Forza) has been developed by 9th Dimension Biotech, Inc., consisting of the lyophilised leaves of the A. Deliciosa plants bioencapsulating recombinant osmotin, with the goal of offering the combined benefits of the osmotin protein in conjunction with the enhanced anti-inflammatory properties of the bioengineered A. Deliciosa plants to consumers. In fact, as recently as in 2016, it has been shown that the bioencapsulation of protein drugs in lyophilized plant cells represents an ideal method of oral protein drug delivery to patients, which surpasses all other methods of protein drug delivery both in safety and efficacy^26^. Therefore, it may be worth exploring the effects of Forza in MS, where Forza may play both anti-inflammatory and neuroprotective effects. Considering the strong need to identify therapies for the progressive forms of the disease, we here propose to explore the effects of Forza in PMS. Moreover, the low impact of current therapies in PMS may not overshadow possible effects of Forza, something that would probably happen with high efficacy treatments in RRMS.

## ETHICAL ASPECTS

After the approval of the Ethic Committees (EC), this study will be conducted in accordance with the study protocol, the current version of the Declaration of Helsinki, applicable Good Clinical Practices (GCP) guidelines and with the specific Italian regulations on non-interventional studies.

Before collecting data, the patients must sign and date the informed consent form having been previously reviewed and approved from the Ethical Committee.

By signing the protocol, the scientific coordinator will be responsible for maintaining the methodological accuracy of the study, both in the design phase and in the evaluation of results.

## STUDY OBJECTIVES

**Primary objective**

The study aims to address the following primary objective:

- Verify the safety of Forza in PMS during the entire treatment period.

**Secondary objectives**

Secondary objectives of the study are:

- Exploring the clinical and biological efficacy of Forza in PMS both after one month and six months of treatment or at the end of the study (in case of early withdrawal).

## METHODS

**Study design**

This is a prospective, multicenter, single-arm non-interventional study. It consists of a first visit six months before the assumption of osmotin (-6M), in which the investigators will inform patients about the study design and objective and the possibility to take part of it.

Then, only after having obtained a written informed consent, demographics characteristics and info about MS history will be collected, and clinical evaluations and assessments will be performed.

During a subsequent baseline visit, six months after the first one, (0M) the same clinical evaluations and assessments will be performed and after their execution the treatment will be administered and taken by the patients.

Two post treatment follow up visits are planned at 1 month (1M) and six months (6M) after the baseline, in which the same clinical evaluations and assessments will be recorded.

Patients meet all inclusion/exclusion criteria will be included in the study after having provided their written informed consent.

They will undergo two pre-treatment assessments: at -6 months (-6M) and at baseline visit (M0) and two post-treatment assessments at month 1 (M1), and at month 6 (M6).

The treatment will consist in the oral administration of Forza capsules at a minimum dosage of 5 grams per day.

At each timepoint the following assessments will be evaluated:

- Electrophysiological (motor evoked potentials – MEPs; somatosensory evoked potentials – SEPs; visual evoked potentials – VEPs)
- Optical coherence tomography (OCT)
- Neurofilaments (NfL)

In addition, clinician assessed outcomes (CAO), patient reported outcomes (PRO) and performance measure (PF) will be acquired for investigating the following domains:

- Motor domain (Expanded Disability Status Scale – EDSS; Timed 25 Foot Walk - T25FW; 12-item Multiple Sclerosis Walking Scale - MSWS12; Nine-Hole Peg Test - 9HPT)
- Cognitive domain (Montreal Cognitive Assessment - MOCA; Symbol Digit Modalities Test - SDMT)
- Emotional domain (Hospital Anxiety and Depression Scale - HADS), and for the bladder domain (Overactive Bladder - OAB).

Brain MRI will also be obtained at each timepoint as standard of care procedure.

In order to investigate the potential effect of Forza on brain metabolism (concentration of glutamate, N-acetylaspartate, creatine, choline) and microstructure, a subgroup of 25 patients (enrolled at the University of Genoa) will undergo MRI with a multi-shell diffusion-weighted (DWI) sequence and proton magnetic resonance spectroscopy (1H-MRI) in addition to routine sequences.

Any changes on disease modifying therapy (DMT), any occurrence of adverse events during treatment period and any interruption of treatment will be recorded on the medical chart.


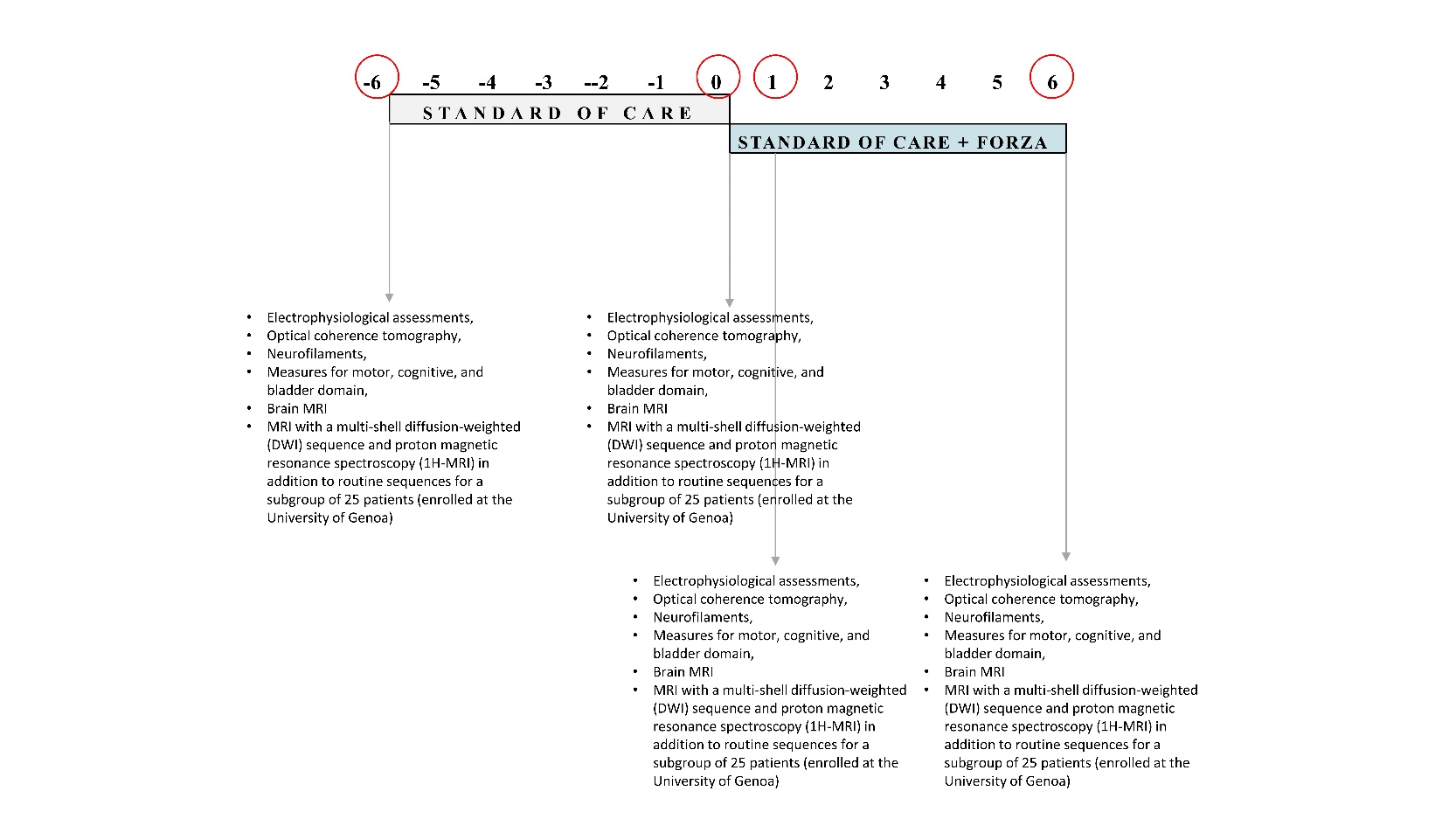


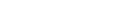


## STUDY TREATMENT

**Forza formulation**

Forza is a nutraceutical supplement provided in capsules, and it consists of lyophilised and pulverised kiwi leaves from bioengineered kiwi (Actinidia Deliciosa) plants overexpressing the tobacco protein Osmotin^6^. Osmotin is a non-toxic plant protein present in all fruits and vegetables, which plays a key role in plants’ stress response and survival. In plants, the overexpression of Osmotin leads to a increased production of flavon-3-ols and other key antioxidant compounds^30^. This plant protein is also a homologue of the human adipokine Adiponectin.

**Forza administration and assumption**

The treatment will consist in the oral administration of a minimum dosage of 5 grams per day of Forza, provided in the form of capsules. Each capsule will contain approximately 0.7g of Forza. The capsules will be administered at the rate of 4 capsules in the morning and 3 additional capsules in the evening for the entire duration of the trial.

**Forza accountability**

Forza is nutraceutical supplement produced at a CGMP facility by 9th Dimension Biotech, Inc. in the United States of America. 9th Dimension Biotech will be accountable for the production and delivery of Forza to FISM for the duration of the trial.

## STUDY POPULATION

**Sites and patients**

The study is planned to be conducted at the MS Centres of the San Martino Hospital in Genoa and of the Sapienza University of Rome.

Patients with a progressive form of MS either untreated or under their current therapy will be enrolled by the MS Centres of the San Martino Hospital in Genoa and of the Sapienza University of Rome.

**Estimated sample size**

A sample of fifty (50) patients guarantee a 90% power to detect a reduction of 20% in the neurofilament change rate (assuming a SD=40%) during the treatment period (6M-1M) as compared to the pre-treatment period (0M – -6M).

**Inclusion and exclusion criteria**

To be included in EOS study, patients must meet all the following inclusion criteria:

- Patients having signed written informed consent
- Males and females aged 18-70 years
- Diagnosis of PMS
- EDSS ≤6.5
- Female patients Not pregnant
- No contraindications to MRI

Exclusion criteria

- HIV positivity
- severe renal, hepatic, oncological, hematological and psychiatric diseases

No data will be collected for patients with even only one of the above-mentioned conditions not respected.

**Early discontinuation of patients**

A subject may withdraw from the study at any time at his/her request, at the discretion of the investigator for safety, behaviour, or administrative reasons.

## DATA MANAGEMENT AND STATISTICAL METHODS

**Data collection and data entry**

All the clinical data will be collected and saved in Red Cap by the personnel from the two centers. Red Cap will be accessible to the Data Management and statistician staff where the data entry, data cleaning and quality control activities will be organized.

Query will be raised if one or more data are unclear or contradictory.

**MedDRA Dictionary**

Medical events reported as Comorbidities and Complications will be classified using the last available version of the Medical Dictionary for Regulatory Activities (MedDRA), an international medical terminology dictionary applied by regulatory authorities and by pharmaceutical industries. The three levels of codes (SOC – PT – LLT) will be implemented into the database.

**Statistical analysis**

All collected data will be analyzed using descriptive statistics. Continuous variables will be described with number of patients with valid observations, mean, standard deviation, median, minimum and maximum value. If necessary, additional descriptive statistics may be calculated (in particular, for non-normally distributed data, the median with interquartile range). Categorical data will be described by frequencies and related percentages within variable. A p-value of 0.05 will be used as the cut off for statistical significance (no statistical test will be used for safety data).

Any comparisons between pre- and post- changes in continuous variables will be evaluated with paired sample t-test or Wilcoxon signed rank-test, as appropriate.

In particular, all safety and efficacy data will be analyzed using descriptive statistics. Safety data will be assessed by reporting and describing all the adverse events. Decisions following the safety pattern will be discussed by the study investigators, but no logic based on statistical test was set.

Depending on the results of interest, a stratified or an exploratory analysis may be conducted.

## PLAN FOR DISSEMINATION OF STUDY RESULTS

Upon completion of data collection and statistical analysis, a final report, including a review of the methods and objectives, together with presentation and discussion of results, will be drawn.

Results of the study will be presented at national and international conferences.

## REFERENCES

1. Dangond F, Donnelly A, Hohlfeld R, Lubetzki C, Kohlhaas S, Leocani L, Ciccarelli O, Stankoff B, Sormani MP, Chataway J, Bozzoli F, Cucca F, Melton L, Coetzee T, Salvetti M. Facing the urgency of therapies for progressive MS - a Progressive MS Alliance proposal. Nat Rev Neurol. 2021 Mar;17(3):185-192. doi: 10.1038/s41582-020-00446-9. Epub 2021 Jan 22. PMID: 33483719.
2. University of California, San Francisco MS-EPIC Team, Cree BAC, Hollenbach JA, Bove R, Kirkish G, Sacco S, Caverzasi E, Bischof A, Gundel T, Zhu AH, Papinutto N, Stern WA, Bevan C, Romeo A, Goodin DS, Gelfand JM, Graves J, Green AJ, Wilson MR, Zamvil SS, Zhao C, Gomez R, Ragan NR, Rush GQ, Barba P, Santaniello A, Baranzini SE, Oksenberg JR, Henry RG, Hauser SL. Silent progression in disease activity-free relapsing multiple sclerosis. Ann Neurol. 2019 May;85(5):653-666. doi: 10.1002/ana.25463. Epub 2019 Mar 30. PMID: 30851128; PMCID: PMC6518998.
3. Kappos L, Wolinsky JS, Giovannoni G, Arnold DL, Wang Q, Bernasconi C, Model F, Koendgen H, Manfrini M, Belachew S, Hauser SL. Contribution of Relapse-Independent Progression vs Relapse-Associated Worsening to Overall Confirmed Disability Accumulation in Typical Relapsing Multiple Sclerosis in a Pooled Analysis of 2 Randomized Clinical Trials. JAMA Neurol. 2020 Sep 1;77(9):1132-1140. doi: 10.1001/jamaneurol.2020.1568. PMID: 32511687; PMCID: PMC7281382.
4. Green AJ. Potential Benefits of Early Aggressive Treatment in Multiple Sclerosis. JAMA Neurol. 2019 Mar 1;76(3):254-256. doi: 10.1001/jamaneurol.2018.4932. PMID: 30644966.
5. Singh NK, Bracker CA, Hasegawa PM, Handa AK, Buckel S, Hermodson MA, Pfankoch E, Regnier FE, Bressan RA. Characterization of osmotin : a thaumatin-like protein associated with osmotic adaptation in plant cells. Plant Physiol. 1987 Oct;85(2):529-36. doi: 10.1104/pp.85.2.529. PMID: 16665731; PMCID: PMC1054289.
6. Rugini, E., Cristofori, V., Martignoni, D., Gutierrez-Pesce, P., Orlandi, S., Brunori, E., Biasi, R., Muleo, R. and Magro, P. (2011). KIWIFRUIT TRANSGENICS FOR OSMOTIN GENE AND INOCULATION TESTS WITH BOTRYTIS CINEREA AND CADOPHORA LUTEO-OLIVACEA. Acta Hortic. 913, 197-203 DOI: 10.17660/ActaHortic.2011.913.25
7. Narasimhan ML, Coca MA, Jin J, Yamauchi T, Ito Y, Kadowaki T, Kim KK, Pardo JM, Damsz B, Hasegawa PM, Yun DJ, Bressan RA. Osmotin is a homolog of mammalian adiponectin and controls apoptosis in yeast through a homolog of mammalian adiponectin receptor. Mol Cell. 2005 Jan 21;17(2):171-80. doi: 10.1016/j.molcel.2004.11.050. Erratum in: Mol Cell. 2005 Feb 18;17(4):611. PMID: 15664187.
8. Carbone F, La Rocca C, Matarese G. Immunological functions of leptin and adiponectin. Biochimie. 2012 Oct;94(10):2082-8. doi: 10.1016/j.biochi.2012.05.018. Epub 2012 Jun 26. PMID: 22750129.
9. Takahashi Y, Watanabe R, Sato Y, Ozawa N, Kojima M, Watanabe-Kominato K, Shirai R, Sato K, Hirano T, Watanabe T. Novel phytopeptide osmotin mimics preventive effects of adiponectin on vascular inflammation and atherosclerosis. Metabolism. 2018 Jun;83:128-138. doi: 10.1016/j.metabol.2018.01.010. Epub 2018 Feb 2. PMID: 29410350.
10. Liu J, Sui H, Zhao J, Wang Y. Osmotin Protects H9c2 Cells from Simulated Ischemia-Reperfusion Injury through AdipoR1/PI3K/AKT Signaling Pathway. Front Physiol. 2017 Sep 25;8:611. doi: 10.3389/fphys.2017.00611. PMID: 28993734; PMCID: PMC5622187.
11. Badshah H, Ali T, Kim MO. Osmotin attenuates LPS-induced neuroinflammation and memory impairments via the TLR4/NFκB signaling pathway. Sci Rep. 2016 Apr 20;6:24493. doi: 10.1038/srep24493. PMID: 27093924; PMCID: PMC4837357.
12. Fantuzzi, G. Adiponectin and inflammation: Consensus and controversy. J Allergy Clin Immunol 2008; 121(2): 326–330.
13. Neumeier M, Weigert J, Schäffler A, Wehrwein G, Müller-Ladner U, Schölmerich J, Wrede C, Buechler C. Different effects of adiponectin isoforms in human monocytic cells. J Leukoc Biol. 2006 Apr;79(4):803-8. doi: 10.1189/jlb.0905521. Epub 2006 Jan 24. PMID: 16434692.
14. Song H, Chan J, Rovin BH. Induction of chemokine expression by adiponectin in vitro is isoform dependent. Transl Res. 2009 Jul;154(1):18-26. doi: 10.1016/j.trsl.2009.04.003. Epub 2009 May 9. PMID: 19524870; PMCID: PMC2727280.
15. Piccio L, Stark JL, Cross AH. Chronic calorie restriction attenuates experimental autoimmune encephalomyelitis. J Leukoc Biol. 2008 Oct;84(4):940-8. doi: 10.1189/jlb.0208133. Epub 2008 Aug 4. PMID: 18678605; PMCID: PMC2638732.
16. Hietaharju A, Kuusisto H, Nieminen R, Vuolteenaho K, Elovaara I, Moilanen E. Elevated cerebrospinal fluid adiponectin and adipsin levels in patients with multiple sclerosis: a Finnish co-twin study. Eur J Neurol. 2010 Feb;17(2):332-4. doi: 10.1111/j.1468-1331.2009.02701.x. Epub 2009 Jun 15. PMID: 19538214.
17. Piccio L, Cantoni C, Henderson JG, Hawiger D, Ramsbottom M, Mikesell R, Ryu J, Hsieh CS, Cremasco V, Haynes W, Dong LQ, Chan L, Galimberti D, Cross AH. Lack of adiponectin leads to increased lymphocyte activation and increased disease severity in a mouse model of multiple sclerosis. Eur J Immunol. 2013 Aug;43(8):2089-100. doi: 10.1002/eji.201242836. Epub 2013 Jun 7. PMID: 23640763; PMCID: PMC3901539.
18. Devorak J, Mokry LE, Morris JA, Forgetta V, Davey Smith G, Sawcer S, Richards JB. Large differences in adiponectin levels have no clear effect on multiple sclerosis risk: A Mendelian randomization study. Mult Scler. 2017 Oct;23(11):1461-1468. doi: 10.1177/1352458516681196. Epub 2016 Dec 7. PMID: 27903934.
19. Çoban A, Düzel B, Tüzün E, Tamam Y. Investigation of the prognostic value of adipokines in multiple sclerosis. Mult Scler Relat Disord. 2017 Jul;15:11-14. doi: 10.1016/j.msard.2017.04.006. Epub 2017 Apr 20. PMID: 28641765.
20. Kvistad SS, Myhr KM, Holmøy T, Benth JŠ, Wergeland S, Beiske AG, Bjerve KS, Hovdal H, Midgard R, Sagen JV, Torkildsen Ø. Serum levels of leptin and adiponectin are not associated with disease activity or treatment response in multiple sclerosis. J Neuroimmunol. 2018 Oct 15;323:73-77. doi: 10.1016/j.jneuroim.2018.07.011. Epub 2018 Jul 24. PMID: 30196837.
21. Signoriello E, Lus G, Polito R, Casertano S, Scudiero O, Coletta M, Monaco ML, Rossi F, Nigro E, Daniele A. Adiponectin profile at baseline is correlated to progression and severity of multiple sclerosis. Eur J Neurol. 2019 Feb;26(2):348-355. doi: 10.1111/ene.13822. Epub 2018 Nov 27. PMID: 30300462.
22. Keyhanian K, Saxena S, Gombolay G, Healy BC, Misra M, Chitnis T. Adipokines are associated with pediatric multiple sclerosis risk and course. Mult Scler Relat Disord. 2019 Nov;36:101384. doi: 10.1016/j.msard.2019.101384. Epub 2019 Sep 5. PMID: 31550559.
23. Signoriello E, Mallardo M, Nigro E, Polito R, Casertano S, Di Pietro A, Coletta M, Monaco ML, Rossi F, Lus G, Daniele A. Adiponectin in Cerebrospinal Fluid from Patients Affected by Multiple Sclerosis Is Correlated with the Progression and Severity of Disease. Mol Neurobiol. 2021 Jun;58(6):2663-2670. doi: 10.1007/s12035-021-02287-z. Epub 2021 Jan 23. Erratum in: Mol Neurobiol. 2021 Feb 18;: PMID: 33486671.
24. Nyirenda MH, Fadda G, Healy LM, Mexhitaj I, Poliquin-Lasnier L, Hanwell H, Saveriano AW, Rozenberg A, Li R, Moore CS, Belabani C, Johnson T, O'Mahony J, Arnold DL, Yeh EA, Marrie RA, Dunn S, Banwell B, Bar-Or A. Pro-inflammatory adiponectin in pediatric-onset multiple sclerosis. Mult Scler. 2021 Feb 1:1352458521989090. doi: 10.1177/1352458521989090. Epub ahead of print. PMID: 33522403.
25. Kwon KC, Daniell H. Oral Delivery of Protein Drugs Bioencapsulated in Plant Cells. Mol Ther. 2016 Aug;24(8):1342-50. doi: 10.1038/mt.2016.115. Epub 2016 Jun 6. PMID: 27378236; PMCID: PMC5023392.
26. Harroud A, Manousaki D, Butler-Laporte G, Mitchell RE, Davey Smith G, Richards JB, Baranzini SE. The relative contributions of obesity, vitamin D, leptin, and adiponectin to multiple sclerosis risk: A Mendelian randomization mediation analysis. Mult Scler. 2021 Feb 19:1352458521995484. doi: 10.1177/1352458521995484. Epub ahead of print. PMID: 33605807.
27. Kim HK, Bae MJ, Lim S, Lee W, Kim S. A Water-Soluble Extract from *Actinidia arguta* Ameliorates Psoriasis-Like Skin Inflammation in Mice by Inhibition of Neutrophil Infiltration. Nutrients. 2018 Oct 2;10(10):1399. doi: 10.3390/nu10101399. PMID: 30279326; PMCID: PMC6213123.
28. Parida S, Siddharth S, Sharma D. Adiponectin, Obesity, and Cancer: Clash of the Bigwigs in Health and Disease. Int J Mol Sci. 2019 May 22;20(10):2519. doi: 10.3390/ijms20102519. PMID: 31121868; PMCID: PMC6566909.
29. Miele M, Costantini S, Colonna G (2011) Correction: Structural and Functional Similarities between Osmotin from *Nicotiana Tabacum* Seeds and Human Adiponectin. PLoS ONE 6(2): 10.1371/annotation/69357261-7e31-40e0-96ff-13cdc783c768.
30. Bhattacharya A, Saini U, Joshi R, Kaur D, Pal AK, Kumar N, Gulati A, Mohanpuria P, Yadav SK, Kumar S, Ahuja PS. Osmotin-expressing transgenic tea plants have improved stress tolerance and are of higher quality. Transgenic Res. 2014 Apr;23(2):211-23. doi: 10.1007/s11248-013-9740-5. Epub 2013 Aug 27. PMID: 23982743.
